# A commentary on “Abbreviated biparametric versus multiparametric MRI for the detection of clinically significant prostate cancer in treatment-naïve patients: a diagnostic test accuracy systematic review and meta-analysis”

**DOI:** 10.1097/JS9.0000000000004515

**Published:** 2025-12-15

**Authors:** Yin Wang, Wenqiang Tang, Yunjiang Zhang

**Affiliations:** Department of Oncology, Santai Hospital Affiliated to North Sichuan Medical College, Mianyang, Sichuan, China

*Dear Editor,*

We read with great interest the diagnostic test accuracy systematic review and meta-analysis by Zhu *et al* comparing abbreviated biparametric MRI (bpMRI) with standard multiparametric MRI (mpMRI) for detecting clinically significant prostate cancer (csPCa) in treatment-naïve patients. The authors synthesize data from 25 studies and report that mpMRI achieves slightly higher pooled sensitivity (0.90 vs 0.87) while maintaining comparable specificity (0.66 vs 0.67), with similar SROC AUCs for both protocols. Their work addresses a highly relevant question in contemporary prostate cancer imaging: whether time- and resource-saving bpMRI can safely replace mpMRI in routine practice.

This study has several important strengths. First, the authors focus specifically on csPCa in treatment-naïve men, aligning their outcome definition with modern ISUP grading (Gleason score ≥3 + 4 in most cohorts), which enhances clinical relevance. Second, the review follows PRISMA-DTA and AMSTAR-2 guidance, is registered in PROSPERO, and uses QUADAS-2 and GRADE to transparently appraise bias and evidence certainty. Third, the statistical approach is robust for diagnostic accuracy research: bivariate random-effects modeling, SROC analysis, and Deeks’ funnel plots are applied, and the authors attempt to dissect heterogeneity through extensive subgroup analyses across study design, *b*-values, magnetic field strength, PI-RADS version, reader experience, and whether analyses were per-patient or per-lesion. Collectively, these efforts provide a comprehensive overview of the current evidence base. However, several methodological and interpretive issues merit cautious consideration.

First, the degree of between-study heterogeneity is substantial. The *I*^2^ values for both sensitivity and specificity exceed 90% for bpMRI and mpMRI, and the overall certainty of evidence is downgraded to “very low” using the GRADE framework. This is broadly consistent with earlier head-to-head meta-analyses, which have also found that bpMRI and mpMRI provide comparable pooled accuracy but with high heterogeneity that precludes firm practice-changing recommendations^[1,2]^. Under such conditions, the pooled point estimates may not reliably reflect performance in any particular clinical setting. We believe this limitation should temper the strength of statements suggesting that the incremental value of DCE is “quite limited” and that bpMRI may act as a straightforward substitute for mpMRI.

Second, the included populations are not entirely homogeneous with respect to the “biopsy-naïve, treatment-naïve” concept. While the authors aimed to focus on men undergoing initial diagnostic work-up, several cohorts included patients with prior biopsies or known prostate cancer, and analysis levels were mixed between per-lesion and per-patient. These factors may inflate diagnostic performance compared with a true screening or first-biopsy population, where lesion conspicuity, tumor volume, and reader confidence can differ. Notably, large single-center cohorts of biopsy-naïve men undergoing bpMRI-guided pathways have reported very high negative predictive values for ruling out csPCa, underscoring the importance of population selection and protocol standardization when extrapolating results^[3]^. In our view, future studies – and ideally individual patient data meta-analyses – should stratify by biopsy-naïve status, previous negative biopsies, and active surveillance cohorts to refine the external validity of bpMRI-based pathways.

Third, publication bias appears to be present for both bpMRI and mpMRI according to Deeks’ tests, yet the clinical implications of this finding are not fully explored. If smaller or negative studies are under-represented, the pooled estimates may overstate diagnostic performance of both protocols and further blur any subtle differences between them. Sensitivity analyses restricted to high-quality, prospective, consecutively enrolled cohorts might help clarify whether bpMRI remains non-inferior when only the most rigorous evidence is considered – a question that recent randomized and prospective non-inferiority trials have begun to address, generally showing bpMRI to be statistically non-inferior to mpMRI for csPCa detection^[4]^.

Fourth, the discussion around DCE-MRI sits at an important but delicate intersection. On the one hand, Zhu *et al* show only a modest sensitivity advantage of mpMRI over bpMRI at the meta-analytic level (0.90 vs 0.87) and appropriately highlight the disadvantages of contrast administration, including longer scan time, cost, gadolinium deposition, and rare but serious adverse events such as nephrogenic systemic fibrosis. On the other hand, prior literature suggests that DCE may meaningfully assist in the assessment of PI-RADS 3 lesions, compensate for suboptimal T2-weighted or diffusion-weighted image quality, and enhance diagnostic confidence for less experienced readers^[1,2]^. In this context, what constitutes an “acceptable” loss of sensitivity for a non-inferiority margin remains a clinical – not purely statistical – judgment. A 3% absolute reduction may be negligible in some settings, yet critical in others (e.g., biopsy triage in young men or those at high genetic risk). Tailored, indication-specific non-inferiority thresholds may therefore be more appropriate than a single global comparison.

Finally, the authors rightly point out the potential economic and workflow advantages of bpMRI, but the present review does not include formal cost-effectiveness analysis or pooled estimates of contrast-related adverse events. Without such data, the statement that bpMRI “may emerge as a viable alternative to the standard MRI protocol” should be interpreted as a hypothesis-generating conclusion rather than a practice-changing recommendation. Prospective multicenter non-inferiority trials – with integrated health economic evaluation and predefined reader-experience strata – will be essential to determine where bpMRI can safely replace mpMRI and where DCE should be maintained as a safeguard. The growing body of RCT and prospective evidence directly comparing bpMRI and mpMRI provides an ideal framework for such evaluations^[4]^.

In summary, Zhu *et al* provide a timely and comprehensive synthesis of the comparative diagnostic performance of bpMRI and mpMRI for csPCa. Their work supports the growing notion that bpMRI can achieve high accuracy in selected settings but also underscores the very low certainty of the current evidence and the persistence of substantial heterogeneity and publication bias. Rather than a binary “replacement” paradigm, we advocate for a more nuanced, phenotype- and context-specific deployment of bpMRI and mpMRI, informed by tumor risk, reader expertise, resource availability, and patient preference.

## Data Availability

All data analyzed or generated during this study are included in the article.
